# In Situ Persulfate Oxidation of 1,2,3-Trichloropropane in Groundwater of North China Plain

**DOI:** 10.3390/ijerph16152752

**Published:** 2019-08-01

**Authors:** Hui Li, Zhantao Han, Yong Qian, Xiangke Kong, Ping Wang

**Affiliations:** 1 Institute of Hydrogeology and Environmental Geology, Chinese Academy of Geological Sciences, Shijiazhuang 050061, China; 2 Key Laboratory of Groundwater Remediation of Hebei Province and China Geological Survey, Shijiazhuang 050061, China

**Keywords:** in situ oxidation, persulfate, injection technology, 1,2,3-trichloropropane, groundwater

## Abstract

In situ injection of Fe(II)-activated persulfate was carried out to oxidize chlorinated hydrocarbons and benzene, toluene, ethylbenzene, and xylene (BTEX) in groundwater in a contaminated site in North China Plain. To confirm the degradation of contaminants, an oxidant mixture of persulfate, ferrous sulfate, and citric acid was mixed with the main contaminants including 1,2,3-trichloropropane (TCP) and benzene before field demonstration. Then the mixed oxidant solution of 6 m^3^ was injected into an aquifer with two different depths of 8 and 15 m to oxidize a high concentration of TCP, other kinds of chlorinated hydrocarbons, and BTEX. In laboratory tests, the removal efficiency of TCP reached 61.4% in 24 h without other contaminants but the removal rate was decreased by the presence of benzene. Organic matter also reduced the TCP degradation rate and the removal efficiency was only 8.3% in 24 h. In the field test, as the solution was injected, the oxidation reaction occurred immediately, accompanied by a sharp increase of oxidation–reduction potential (ORP) and a decrease in pH. Though the concentration of pollutants increased due to the dissolution of non-aqueous phase liquid (NAPL) at the initial stage, BTEX could still be effectively degraded in subsequent time by persulfate in both aquifers, and their removal efficiency approached 100%. However, chlorinated hydrocarbon was relatively difficult to degrade, especially TCP, which had a relatively higher initial concentration, only had a removal efficiency of 30%–45% at different aquifers and monitoring wells. These finding are important for the development of injection technology for chlorinated hydrocarbon and BTEX contaminated site remediation.

## 1. Introduction

After decades of high-speed economic development, the chemical industry has released a large volume of miscellaneous chemicals into soil and groundwater in China [1,2,3]. A survey on groundwater pollution in the North China Plain showed that the detection rate of organic pollutants in groundwater samples was nearly 40%; in particular, chlorinated hydrocarbons and aromatic hydrocarbons account for 30% and 25% of the total organic pollutants, respectively [4]. A combination of these two kinds of pollutants is present in a large number of contaminated sites, requiring special attention due to their high risk to human health.

Highly toxic 1,2,3-trichloropropane (TCP) is a recalcitrant chlorinated hydrocarbon. Soil and groundwater contamination with TCP occurs at industrial and agricultural sites due to its use as a solvent for degreasing, as a feedstock for polymer production, and as a precursor to some soil fumigants [5]. Furthermore, TCP has been detected in hundreds of surface water and drinking water sources at levels of 0.1–100 μg/L in the United States [6,7,8]. In China, TCP is an emerging contaminant because of increased recognition of its occurrence in groundwater. In some of these sites, groundwater contaminated by TCP poses high risk to the health of local residents and will continue to do so for decades if no remediation measures are taken.

For organic pollutants with poor solubility, in situ treatment measures were considered to be more cost effective than pump and treat [9]. In situ reduction, in situ oxidation, and in situ biological degradation are widely used to degrade organic contaminants in groundwater, but for TCP, some studies have proved that nanoscale zero-valent irons (NZVIs) had little effect on TCP dechlorination and that nanoscale iron sulfide and nanoscale zero-valent zincs (NZVZs) were effective but with a larger dosage of 250 g/L [5,10]. Studies on TCP biodegradation also found that no known natural organisms could use TCP as a carbon source for growth under aerobic conditions, and the anaerobic degradation rate was almost negligible [11,12]. In situ chemical oxidation (ISCO) might be effective but has not been reported so far.

As a common remediation technology, ISCO can effectively degrade most organic pollutants or decrease their toxicity by adding chemical agents directly into contaminated soil or groundwater [13,14]. Oxidants used in ISCO include potassium permanganate, Fenton’s reagent and ozone [15,16], and persulfate [17,18,19,20]. The persulfate ion (S_2_O_8_^2−^) has a standard redox potential of 2.01 V (Equation (1)), which is close to that of ozone (2.07 V) and higher than that of permanganate (1.68 V) and hydrogen peroxide (1.70 V) [21]. The oxidation of persulfate on recalcitrant pollutants such as TCP is relatively weak when it is not activated. However, persulfate can be activated and decomposed to sulfate radical anion (SO_4_^−^·) with the help of heat, light (UV), ultrasound, and transition metal ions (e.g., Mn^+^, M = Fe^2+^, Ag^+^, Ce^2+^, and Co^2+^) [22,23,24,25]. SO_4_^−^· has one lone pair electron with a redox potential of 2.6 V (Equation (2)), which is much higher than that of S_2_O_8_^2−^ and close to that of the hydroxyl radical (·OH) (2.8 V) [26]. Therefore, SO_4_^−^· has a strong oxidation ability which can probably degrade recalcitrant organic pollutants such as TCP. Our previous study has testified TCP could be degraded by Fe^2+^-activated persulfate in the laboratory [27], but no field application of activated persulfate for TCP degradation was reported so far.

(1) $$S_{2}O_{8}{}^{2-}+2e^{-}\overset{}{\to }2SO_{4}{}^{2-}$$

(2) $$SO_{4}{}^{-}\cdot +e^{-}\overset{}{\to }SO_{4}{}^{2-}$$

Previous studies have testified persulfate can last a long time in soil, which can provide more time for the transport of persulfate to contact and oxidize organic compounds [28]. For examples, in FMC Corporation, patent persulfate activation technology has been applied to 300 sites in 35 of the US states for the remediation of soil and groundwater contaminated by petroleum-based hydrocarbons, methyl tert-butyl ether (MTBE), polycyclic aromatic hydrocarbons (PAHs), polychlorinated biphenyls (PCBs), chlorinated alkanes, and alkenes. In North Carolina, a site contaminated by BTEX, MTBE, and naphthalene (210–3200 µg/L) could not be remediated through continuous gas injection over two years but was degraded to below the detection limit after only 40 h by an injection of 1250 L 8.8% persulfate with hot steam. In California, the total volatile organic compounds (VOCs) in the groundwater of Eastland Woolen Mill reached as high as 100–10,000 μg/L. From 2003 to 2006, the persulfate oxidation technology was implemented at different scales, and the results showed that 90% and 60% of the contaminants in the groundwater and the soil were degraded, respectively. Similarly, to degrade dichloromethane in the aquifer at a contaminated site in Los Angeles, 7250 kg of persulfate was injected into groundwater through 17 wells within 22 days, and the concentration of dichloromethane in six wells decreased by 94%–99% in 120 days. Based on previous studies, activated persulfate was believed to be effective for in situ oxidation of TCP in groundwater. In this study, laboratory and in situ tests were carried out to explore the effectiveness of persulfate oxidation on TCP remediation, which would provide a significant technical reference for the remediation of TCP-contaminated sites.

## 2. Materials and Methods

### 2.1. Description of the Site

**Figure 1:**
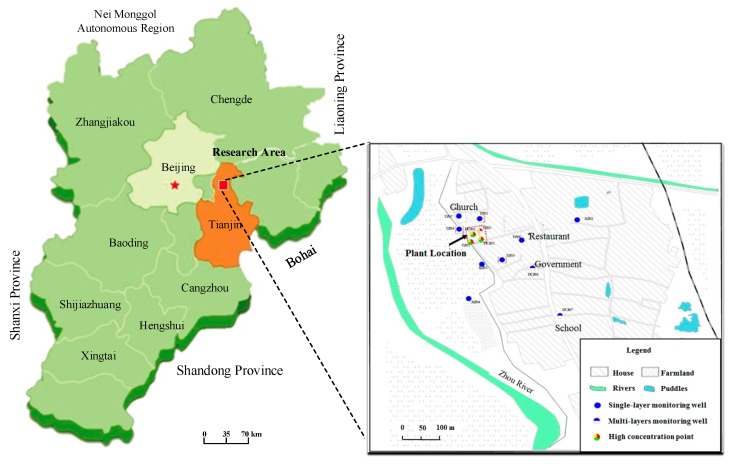
Location and pollution survey points of the site.

The TCP contaminated site is located in the northwest of the North China Plain (Figure 1). The terrain of the site is generally flat, and the strata are mainly composed of Quaternary alluvial and fluvial deposits. The pollutants were leaked from a small-scale chemical plant operated during 1976–1980, in which waste chemicals containing chlorinated solvents and BTEX were stored in a tank without a waterproof lining, the solvents were leaked into soil and groundwater, and the contamination plume in the groundwater extended to around 600 m downstream.

The shallow strata within 40 m of the site are mainly composed of silt, medium sand, coarse sand, and clay. The average depth of the shallow groundwater table in this site is about 4–7 m, and the general flow direction of groundwater is from northwest to southeast. The hydraulic gradient is approximately 0.1‰, and the average flow velocity of groundwater is 0.48 cm/day. Shallow groundwater is mainly recharged by rainfall infiltration and the leakage of the upstream river and discharged by evaporation and pumping wells. The annual variation of groundwater level is around 1–4 m.

Organic contaminants and other chemical compositions of the groundwater were measured regularly for two years. The pollutants were mainly distributed in the shallow aquifer of 6–18 m. Several chlorinated hydrocarbons and aromatic hydrocarbons including TCP, 1,2-dichloropropane, 1,3-dichloropropane, and BTEX with a concentration range of 100–10,000 μg/L have been recorded. TCP is the principal pollutant and its maximum concentration reached 30,164 μg/L in the pollution source zone before the remediation test. One of the detailed chemical compositions of groundwater in the source zone is shown in Table 1.

### 2.2. Bench-Top Tests for the Degradation of TCP by Activated Persulfate

According to our previous study [27], citric acid could better chelate with Fe^2+^ than ethylene diamine tetraacetic acid (EDTA) resulting in a better removal effect of TCP by activated persulfate. Moreover, when the molar ratio of S_2_O_8_^2^^−^, Fe^2+^, and citric acid was 20:1:1, 20:5:1, and 100:5:1, the increase of the TCP degradation rate was limited [29]. Therefore, citric acid was chosen as the chelating agent and a 20:5:1 molar ratio of S_2_O_8_^2−^, Fe^2+^, and citric acid was applied in this test. Laboratory tests were conducted in 150 mL Erlenmeyer flasks. Firstly, according to the specified proportion, a calculated amount of Na_2_S_2_O_8_, FeSO_4_⋅7H_2_O, and citric acid were mixed in deionized water to prepare the oxidant solution. Secondly, 1.25 mL of 24.0 g/L TCP stock solution prepared with methanol was poured into the flasks with 100 mL oxidant solution (the molecular ratio of S_2_O_8_^2−^ and TCP was 20:1), and then the flasks were shaken in an oscillator and the residual concentration of TCP was measured at specific time. To investigate the consumption of oxidant by aromatic hydrocarbon and organic matter in the aquifer matrix, another two groups of tests under the same condition were carried out, but with 1.00 mL 3.0 g/L benzene stock solution and 20 g dry aquifer matrix collected by drilling machine being added to each of the flasks, respectively. The concentrations of TCP and benzene in the flasks were measured by gas chromatograph (Shimadzu GC2010 Plus, Kyoto, Japan) equipped with a DB-624 capture column and a flame ionization detector (FID).

### 2.3. In Situ Remediation by Injection of Persulfate Solution

**Figure 2:**
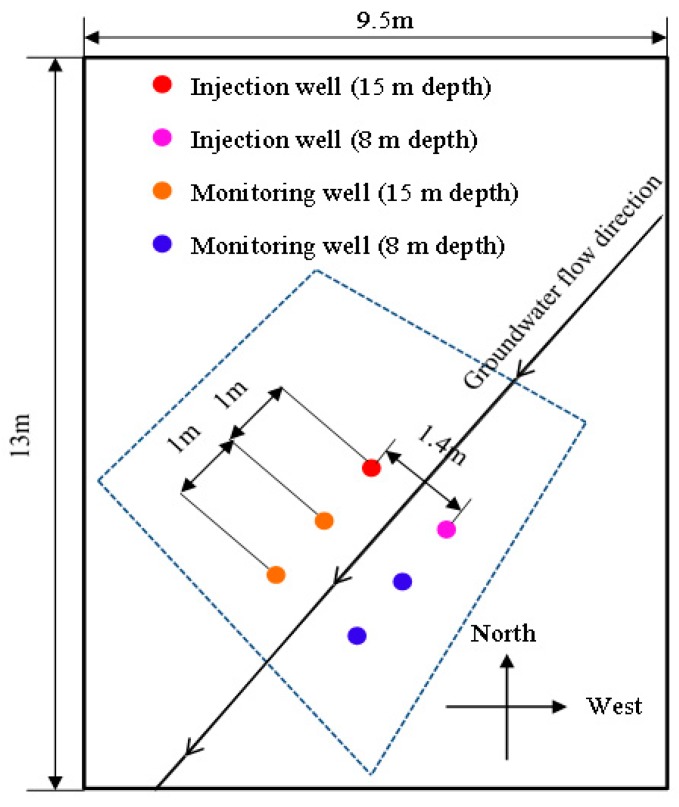
Distribution of injection wells and monitoring wells.

In situ injection of persulfate solution was carried out for aquifers at the depth of 8 and 15 m, there is a silt and fine sand layer of 1 m thickness between these two aquifers at this site. One injection well and two monitoring wells were built up along a straight line in the direction of the groundwater flow. The distances between the injection wells and downstream monitoring wells and between the monitoring wells were all 1.0 m. The horizontal distance between the injection wells was 1.4 m (Figure 2).

The oxidant solution used for the field test contains 1.68 mol/L persulfate, 0.43 mol/L ferrous sulfate, and 0.21 mol/L citric acid. First, 12 kg of ferrous sulfate and 4 kg citric acid were dissolved into 100 L tap water collected from the houses of local residents and were stirred by pneumatic agitator constantly. Then 40 kg of persulfate was added to the mixed solution, the color of the solution was changed from yellowish-green to dark brownish-red simultaneously, which indicated the oxidation of Fe(II) to Fe(III) and the activation of persulfate.

A pneumatic diaphragm pump driven by air compressor was used to inject the oxidant solution into the aquifer through two injection wells. The output pressure of the air compressor was kept at 0.3–0.5 MPa and the average injection rate was 480 L/h. For the aquifer of 15 m, oxidant solution was injected at an output pressure of 0.85 MPa and an average rate of 400 L/h. The injection volume of both layers was 6 m^3^.

### 2.4. Sampling and Analysis of Groundwater Parameters

Groundwater samples were collected through injection and monitoring wells by peristaltic pumps at regular time intervals. The samples for organic measurement were collected in 40 mL brown headspace vials containing 1.5 mL of 6 M HCl with one duplicate sample, and those for inorganic measurement were collected in 500 mL plastic bottles. A AHQ30D portable water quality analyzer (HACH, Loveland, USA) was used for field measurement for pH, oxidation–reduction potential (ORP), and electrical conductivity (EC). Their limits of detection and calibration range were 0.01, 0.1 mV, 1 μs/cm, and ±0.02, ±1.0 mV, ±1 μs/cm, respectively. The titrimetric method by acid standard solution was used for HCO_3_^−^ and CO_3_^2^^−^ analysis. Ion chromatography (Metrohm, 883, Herisau, Switzerland) was used for K^+^, Ca^2+^, Na^+^, Mg^2+^, Cl^−^, and NO_3_^−^ measurement. The barium chromate spectrophotometric method was used for SO_4_^2^^−^ analysis by a spectrophotometer (Beifen-Ruili, Vis-7220N, Beijing, China). Fe and Mn were measured by an atomic absorption spectrophotometer (PerkinElmer, AAnalyst 800, Waltham, USA). The detection limit of inorganic components was 0.01 mg/L. Organic contaminants were analyzed by a purge-trap/gas chromatograph mass spectrometer (P&T/GC–MS, Thermo Fisher Scientific, ISQTM 7000, Waltham, USA) equipped with a DB-624 capillary column. The groundwater samples were purged with high-purity N_2_ at 35 mL/min for 10 min, then the trapped pollutants were desorbed by a 250 °C cartridge of P&T concentrator with a He gas flow at 5 mL/min. The GC temperature program was set as follows: The oven temperature was initially held at 35 °C for 2 min, increased to 150 °C at a rate of 8 °C/min and held for 4 min, and increased to 250 °C at a rate of 6 °C/min and held for 3 min. The samples were injected using a split ratio of 5:1. The temperatures of the transfer line and ion source were set to 230 and 250 °C, respectively. The detection limits of TCP, 1,2-dichloropropane, 1,3-dichloropropane, trichloromethane, 1,2-dichloroethane, benzene, methylbenzene, ethylbenzene, and o-xylene were all 0.5 μg/L, those of dichloromethane and propanone were both 50 μg/L, and those of m-xylene/p-xylene and chloromethane were 1.0 and 5.0 μg/L, respectively.

## 3. Results and Discussion

### 3.1. Degradation of TCP by Persulfate Solution in Deionized Water

**Figure 3:**
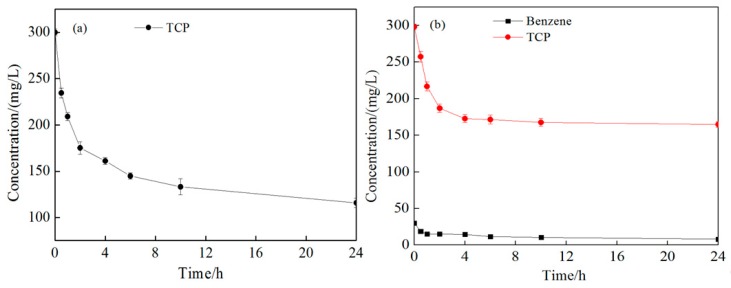
Changes of 1,2,3-trichloropropane (TCP) and benzene concentration in deionized water. Conditions: (**a**) The experiment was conducted with single contaminate of 280.64 mg/L TCP, 9.69 g/L persulfate, 20:5:1 molar ratio of S_2_O_8_^2^^−^, Fe^2+^, and citric acid at ambient temperature; (**b**) the experiment was conducted with mixed contaminates of 296.31 mg/L TCP and 28.32 mg/L benzene, 9.69 g/L persulfate, 20:5:1 molar ratio of S_2_O_8_^2^^−^, Fe^2+^, and citric acid at ambient temperature.

Figure 3a shows the degradation of TCP by persulfate in deionized water. TCP concentration declined from 280.64 to 115.80 mg/L in 24 h, which indicates a removal efficiency of 61.4%. Gu et al. [30] used persulfate activated with Fe(III)-ethylenediamine disuccinic acid (EDDS) complex for trichloroethylene (TCE) degradation, and they found that, when the molar concentration ratio of persulfate to TCE was 100:1, the removal of TCE was in the range of 85% to 96%. Miao’s [31] study on perchloroethylene (PCE) oxidation by alkali-activated persulfate proved that PCE could be almost removed in 24 h. Similarly, the research from Du and Zhang verified that the removal effect of p-chloroaniline by persulfate approached 72% in 24 h when at pH 7 and the molar concentration ratio of persulfate and p-chloroaniline was 50:1 [32]. Compared with these studies, in our test, a smaller molar ratio of persulfate to pollutant was adopted for cost efficiency. Furthermore, citric acid was added as a chelating agent, which caused a decrease in pH. Therefore, the lower degradation rate was due not only to the TCP’s recalcitrance but also to the results of the lower persulfate concentration and lower pH in the reaction system. Figure 3b shows the degradation of benzene and TCP under coexistence conditions. Benzene concentration declined from 28.32 to 8.13 mg/L with a removal efficiency of 71.3%, and TCP concentration declined from 286.31 to 164.89 mg/L with a removal efficiency of 42.4% in 24 h, which indicates that benzene is easier to degrade by persulfate than TCP. Compared with the result in Figure 3a, TCP degradation efficiency declined by 30.9% due to benzene’s competitive consumption of persulfate. Therefore, more persulfate should be added to ensure TCP degradation effectively under the coexistence condition of other organics such as BTEX.

**Figure 4:**
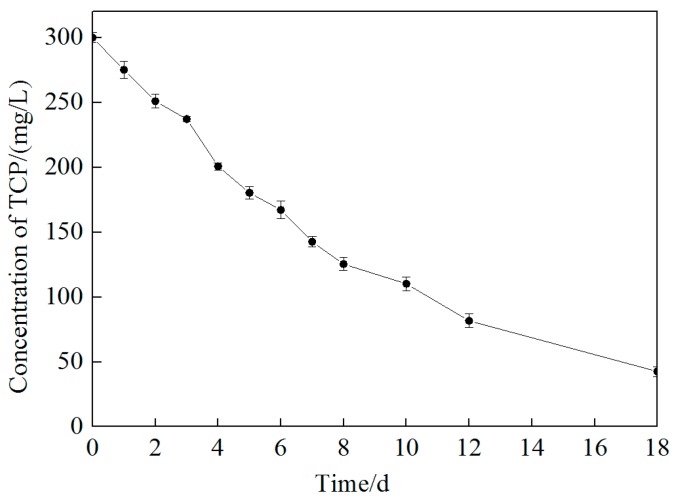
Change of TCP concentration in deionized water experiment conducted with 20 g soil, 300 mg/L TCP, 9.69 g/L persulfate, and 20:5:1 molar ratio of S_2_O_8_^2^^−^, Fe^2+^, and citric acid at ambient temperature.

When soil was added to the same reaction system, the degradation rate of TCP was sharply reduced. The concentration of TCP decreased to 275.20 mg/L in 24 h, equal to a removal efficiency of only 8.3%. However, TCP concentration continued to drop and nearly 85.5% of TCP was removed after 18 days (Figure 4). This indicates that the presence of the aquifer matrix retarded the rate but did not diminish the effectiveness of TCP degradation, as about the same degradation ratio was achieved when a soil matrix was presented. The retardation mechanism by soil might be due to the adsorption of S_2_O_8_^2−^, Fe^2+^, or citric acid chelated Fe^2+^, and also the SO_4_^−^ radical. A test by Liang et al. [33] using S_2_O_8_^2−^ and TCE molar ratios of 5:1 and 10:1 resulted in 52% and 74% of persulfate remaining after a 24 h period, respectively. This suggested that a higher initial concentration of persulfate might result in a higher percentage of unused persulfate, which could be used to oxidize intermediate products left. The TCP degradation process can be expressed by a pseudo-first-order kinetic equation. The overall rate constant and half-life of the reaction were 0.11 day^−1^ and 6.3 days with the presence of soil, respectively.

### 3.2. Products of TCP Oxidation by Activated Persulfate

**Figure 5:**
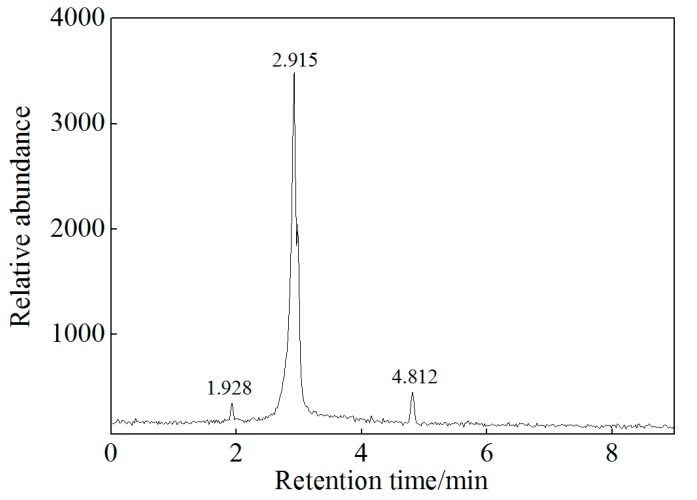
Chromatogram of oxidation products of TCP.

A few previous studies [10,34,35,36] provided detailed information on the pathways of TCP oxidation as follows: TCP was initially oxidized and hydrolyzed to chloropropanol, dichloro-propenes, and chloropropanol (as intermediates), then oxidized to 1,3-dichloro-propanone, chloroacetic acid, and formic acid. Therefore, the oxidation process accompanies hydrolysis. In this study, 6 μg/L chloromethane, 1350 μg/L propanone, and 3.2 μg/L 1,2-dichloroethane were detected in 1.928, 2.915, and 4.812 min, respectively (Figure 5). Propanone was the intermediate product of TCP oxidation. Under reduce conditions, hydrogenolysis of TCP is not expected to be facile but is possible. Organic acid or alcohol can be used as a hydrogen donor in hydrogenolysis reaction [37]. Therefore, a small amount of TCP was decomposed by citric acid resulting in the production of chloromethane and dichloroethane.

### 3.3. Results of In Situ Oxidation Tests

#### 3.3.1. Change of Groundwater Chemistry

**Figure 6:**
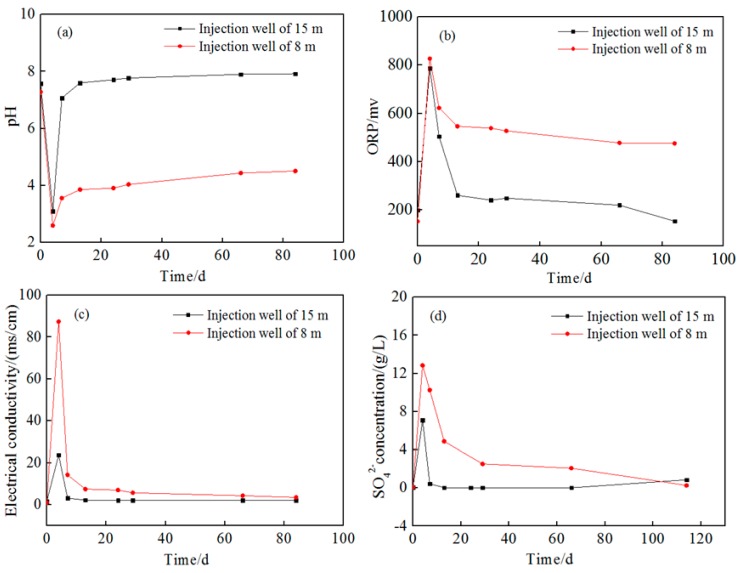
Change of geochemical parameters at injection wells. (**a**) pH; (**b**) oxidation–reduction potential (ORP); (**c**) electrical conductivity; (**d**) the concentration of SO_4_^2−^.

The change of pH, ORP, electrical conductivity, and SO_4_^2−^ concentration of groundwater in injection wells are shown in Figure 6. The pH rapidly decreased to 2.52–3.01 and the ORP increased to over 800.3 mV at injection wells in the first five days following injection. Moreover, the concentration of SO_4_^2^^−^ and the electrical conductivity of groundwater in both layers increased significantly, indicating the presence of oxidation solution. After five days, the pH and ORP changed back gradually because of the progress of the redox reaction. However, unlike that in the 15 m depth well, the pH and ORP in the 8 m depth well did not resume their initial state even after 85 days. This result may be attributed to the different groundwater flow rates in different aquifer media and the different amount of residual citric acid. Furthermore, the injected solution was diluted to different ratios in aquifers with different thickness—where as the 8 m deep aquifer was about 3.5 m thick, the 15 m deep one was 7.5 m thick. SO_4_^2−^ and EC also rapidly changed back after injection due to dilution but then slowly changed because SO_4_^2^^−^ was generated as a reduction product.

**Figure 7:**
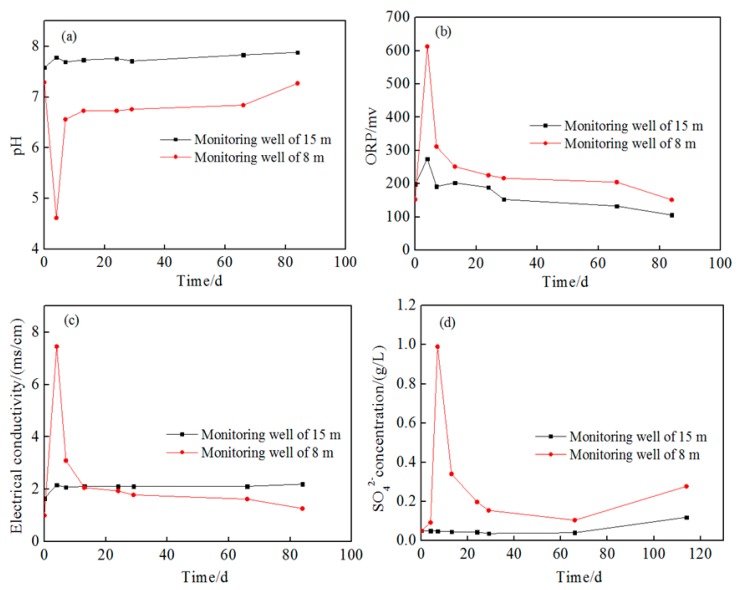
Change of geochemical parameters at monitoring wells 2 m downstream. (**a**) pH; (**b**) oxidation–reduction potential (ORP); (**c**) electrical conductivity; (**d**) the concentration of SO_4_^2−^.

The trends of pH, ORP, electrical conductivity, and SO_4_^2−^ concentration of groundwater at 1 m downstream wells are shown in the Figure S1 in Supporting Information, and those at 2 m downstream are shown in Figure 7. It can be seen that the parameters in the 15 m depth of aquifer still showed slighter change than those in the 8 m depth aquifer. On the other hand, the parameters’ variation at the monitoring well of 8 m were consistent with that in the injection well, indicating that the oxidant had transported more than 2 m. From Figure 6 and Figure 7, it can be seen that the change of parameters at the monitoring wells was smaller than that at the injection wells, suggesting a concentration gradient of oxidant along the groundwater flow direction. Therefore, it is estimated that the degradation of pollutants gradually decreased along the groundwater flow.

Except ORP, no observable change of pH and SO_4_^2−^ concentration happened during the whole observation period, which might also be attributed to the strong dilution effect in 15 m depth and natural effect of the aquifer matrix for pH, but the rise of ORP and EC indicates that the persulfate solution also transported to this well.

#### 3.3.2. Change of Pollutant Concentration

**Figure 8:**
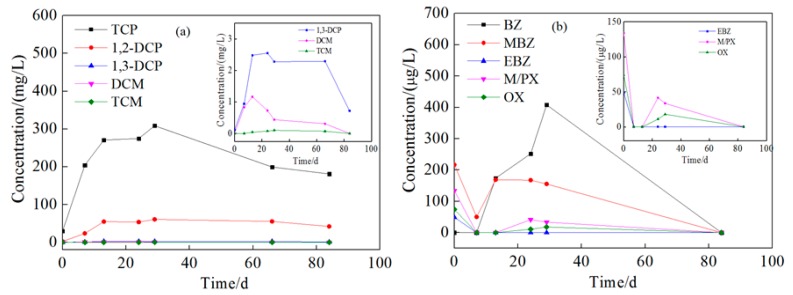
Concentration change of pollutants at shallow injection well (1,2,3-trichloropropane, 1,2-dichloropropane, 1,3-dichloropropane, dichloromethane, trichloromethane, benzene, methylbenzene, ethylbenzene, m-xylene/p-xylene, and o-xylene are abbreviated as TCP, 1,2-DCP, 1,3-DCP, DCM, TCM, BZ, MBZ, EBZ, M/PX, and OX, respectively). Ordinate: (**a**) The concentration of chlorinated hydrocarbons; (**b**) the concentration of benzene, toluene, ethylbenzene, and xylene (BTEX). Inset: (**a**) Amplified concentration change curves of 1,3-DCP, DCM, and TCM; (**b**) amplified concentration change curves of EBZ M/PX, and OX.

**Figure 9:**
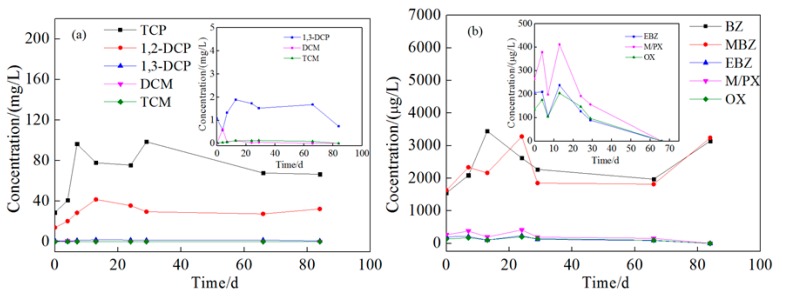
Concentration change of pollutants at deep injection well. Ordinate: (**a**) The concentration of chlorinated hydrocarbons; (**b**) the concentration of BTEX. Inset: (**a**) Amplified concentration change curves of 1,3-DCP, DCM and TCM; (**b**) amplified concentration change curves of EBZ, M/PX, and OX.

**Figure 10:**
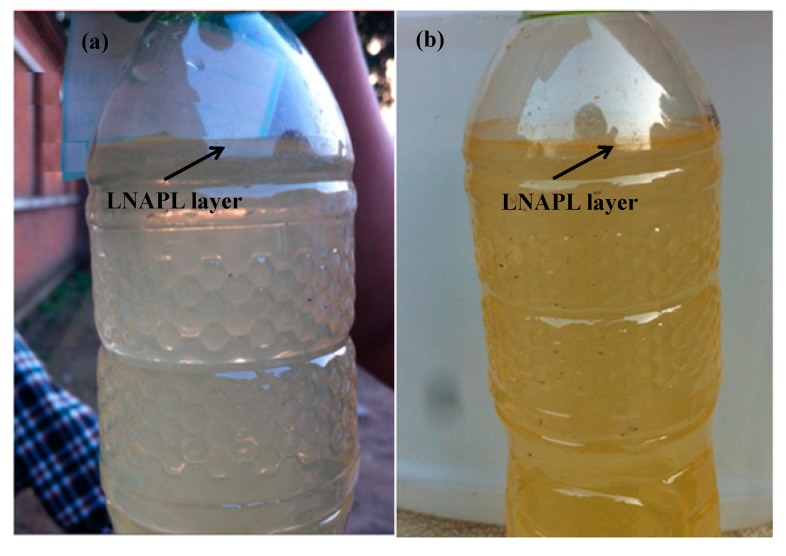
Light non-aqueous phase liquid (LNAPL) in groundwater samples at monitoring wells. (**a**) Groundwater from 8 m depth aquifer; (**b**) groundwater from 15 m depth aquifer.

The changes of pollutants at injection wells at 8 and 15 m depth are shown in Figure 8 and Figure 9. In the process of injection, the concentration of all pollutants continuously increased, except for that of aromatic hydrocarbons in 8 m depth well, which might be due to the disturbance and dissolving of non-aqueous phase liquid (NAPL) pollutants in the aquifer, which have been previously observed in many groundwater remediation projects [38]; the presence of light non-aqueous phase liquid (LNAPL) has been observed in the groundwater samples collected at this site to support this speculation (Figure 10). The concentration of pollutants in groundwater increased for 25–30 days, and then began to decrease. Over the next 55 days, in the 8 m depth aquifer, the concentration of TCP, 1,2-dichloropropane, and 1,3-dichloropropane decreased from 308.54, 60.72, and 2.55 mg/L to 181.10, 42.20, and 0.72 mg/L with a removal efficiency of 41.3%, 34.1%, and 71.8%, respectively. The BTEX concentration in the 8 m depth aquifer decreased in the first seven days during the injection due to the dilution of persulfate solution, and then increased for another 22 days. This might be due to the intrusion of high concentration of BTEX at the 15 m depth aquifer during the injection process. After 85 days, the concentration of BTEX decreased to below the detection limit, and the removal efficiency approached 100%. In the 15 m depth aquifer, TCP, 1,2-dichloropropane, and 1,3-dichloropropane decreased from 96.44, 41.68, and 1.89 mg/L to 66.52, 27.41, and 0.74 mg/L with a removal efficiency of 31.0%, 34.2%, and 60.8%, respectively. The concentration of benzene and methylbenzene decreased during 28–68 days after the injection but increased again on the 85th day, maybe due to further NAPL dissolution resulting from sampling disturbance.

In both the 8 and 15 m depth aquifers, the removal efficiencies of dichloromethane, trichloromethane, ethylbenzene, xylene, and o-xylene all approached 100%, and that of TCP, 1,2-dichloropropane, and 1,3-dichloropropane reached 31%–72%. If we calculated the degradation rate based on their increased concentration after NAPL dissolution, the degradation rates of benzene and methylbenzene were about 45%–100%. These results suggest that the remediation effect of persulfate to pollutants was not affected by the depth of aquifer but was connected with the degradability and concentration of pollutants in the groundwater. The overall removal efficiency for aromatic hydrocarbons was higher than that for chlorinated hydrocarbons, and TCP was especially much more difficult to oxidize, which could be attributed to its stable chemical structure and higher initial concentration. Moreover, when there is NAPL in the aquifer, the concentration of organic pollutants will tend to decrease with some fluctuations.

**Figure 11:**
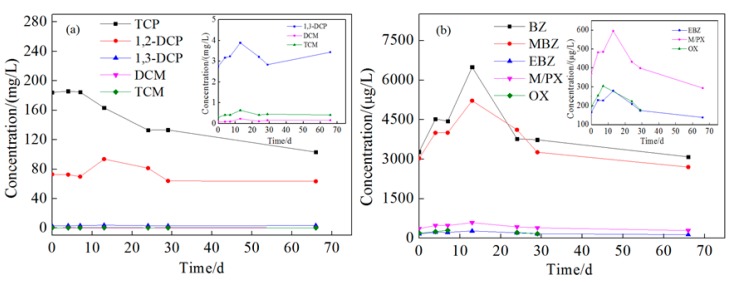
Concentration changes of pollutants at the shallow monitoring well 2 m downstream. Ordinate: (**a**) The concentration of chlorinated hydrocarbons; (**b**) the concentration of BTEX. Inset: (**a**) Amplified concentration change curves of 1,3-DCP, DCM, and TCM; (**b**) amplified concentration change curves of EBZ, M/PX, and OX.

**Figure 12:**
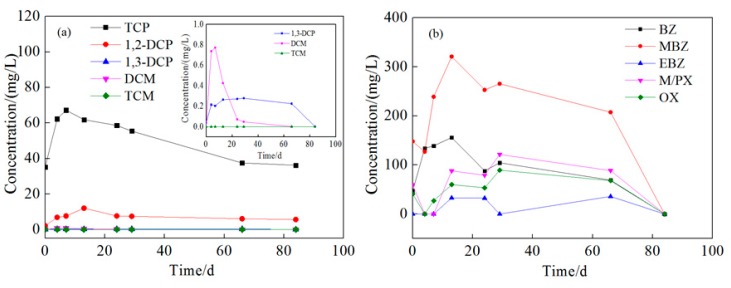
Concentration changes of pollutants at the deep monitoring well 2 m downstream. Ordinate: (**a**) The concentration of chlorinated hydrocarbons; (**b**) the concentration of BTEX. Inset: (**a**) Amplified concentration change curves of 1,3-DCP, DCM, and TCM.

Compared with the injection wells, the change of pollutants at monitoring wells was more complicated. This is due to the effect of oxidation by reagent, groundwater flow, and NAPL dissolution. The concentration changes of pollutants at 1 m downstream wells are shown in the Figures S2 and S3 in Supporting Information, and those at 2 m downstream are shown in Figure 11 and Figure 12. The tendency of contaminant concentration changes was about the same in 1 and 2 m downstream wells but with a smaller extent in the 2 m downstream wells. In the 2 m downstream wells, for chlorinated hydrocarbon in both aquifers, the concentration increased to different extents in the first 15 days which might be due to the flow through of high-concentration pollutants from upstream. Afterward, the pollutant concentration gradually decreased, which could be attributed to the oxidation reaction but not groundwater flow because the higher concentration of pollutants of upstream remained for 10–30 days. The removal rates of TCP were 44.5% and 46.1% and those of 1,2-dichloropropane were 32.2% and 53.41%. 1,3-dichloropropane, dichloromethane, and trichloromethane were all degraded to below detection limit. For BTEX in both aquifers, the concentration increased in the first 30 days. Comparing Figure 8, Figure 9, Figure 11 and Figure 12, it can be found that the concentration of BTEX at the monitoring wells was much higher than that at the injection wells. This is because the initial concentration of BTEX in the monitoring wells was higher than that in the injection wells. As shown in Figure 10, clear LNAPL layers can be observed in the samples of both aquifers, and the LNAPL layer at 15 m was thicker than that at 8 m. Accelerated groundwater flow during the injection process caused NAPL dissolution and then BTEX concentration increased. In the following 55 days, BTEX concentration decreased by dilution and oxidation effect. The removal efficiencies of benzene and methylbenzene in the 8 m aquifer were 55.8% and 48.26%, respectively, and the remaining BTEX in both aquifers was removed by 100%.

In summary, activated persulfate could effectively degrade chlorinated hydrocarbons including TCP, 1,2-dichloropropane, 1,3-dichloropropane, dichloromethane, trichloromethane, and BTEX in groundwater simultaneously. The degradation rate of chlorinated hydrocarbons was lower than that of BTEX, maybe due to their different degradability and initial concentrations. The oxidative degradation process could extend to about 90 days.

## 4. Conclusions

To investigate the remediation efficiency of oxidation with activated persulfate for chlorinated hydrocarbons, especially TCP in groundwater, an in situ injection experiment was conducted in a contaminated site with high concentrations of TCP, 1,2-dichloropropane, 1,3-dichloropropane, dichloromethane, trichloromethane, and some BTEX. The changes in pH, ORP, electrical conductivity, and SO_4_^2−^ concentration during the injection process were described in detail, as well as the removal efficiency of pollutants in groundwater. The research findings are summarized as follows: In bench-top tests, when the molar ratio of S_2_O_8_^2−^ to TCP was 20:1, the removal efficiency of TCP was 61.4% in 24 h. Aquifer material retarded the TCP degradation, and the removal efficiency was only 8.3% in 24 h. The overall rate constant and half-life of the reaction were 0.11 day^−1^ and 6.3 days, respectively. In the field test, the oxidation reaction caused a decrease of pH, and an increase of ORP, electrical conductivity, and SO_4_^2−^ concentration, especially in the 8 m depth aquifer, which indicated that the extent of the reaction was influenced by the thickness of the aquifer due to their different dilution ratios of injected oxidant. The change of these parameters at the monitoring wells was smaller than that at the injection wells due to the concentration gradient of oxidant along the groundwater flow direction. The concentration of pollutants increased due to the dissolution of NAPL resulting from hotspot flushing at the initial stage, and then decreased for persulfate oxidation. The overall removal efficiency for aromatic hydrocarbons was higher than that for chlorinated hydrocarbons. At injection wells, in both 8 and 15 m aquifers, the removal efficiencies of ethylbenzene, xylene, and o-xylene all approached 100%, and those of benzene and methylbenzene were about 45%–100%. The removal efficiency of TCP, 1,2-dichloropropane, and 1,3-dichloropropane reached 31%–72%. The results suggest that the remediation effect of persulfate to pollutants was not affected by the depth of the aquifer but was connected with the degradability and concentration of the pollutants. The concentration change of pollutants at the monitoring wells was also influenced by the concentration of the oxidant and groundwater flow. The removal rates of TCP were 44.5% and 46.1%, and those of 1,2-dichloropropane were 32.2% and 53.41%, respectively. 1,3-dichloropropane, dichloromethane, and trichloromethane were all degraded to below the detection limit. The removal efficiencies of benzene and methylbenzene in the 8 m aquifer were 55.8% and 48.26%, respectively, and the remaining BTEX in both aquifers was removed by 100%.

## Figures and Tables

**Table 1:** Chemical analysis of groundwater in the pollution source zone.

Organic Pollutants	Concentration (mg/L)	Inorganic Components	Concentration (mg/L)
1,2-dichloropropane	13.95	K^+^	2.87
1,3-dichloropropane	1.08	Na^+^	66.85
1,2,3-trichloropropane	28.76	Ca^2+^	114.20
trichloromethane	0.05	Mg^2+^	34.56
dichloromethane	0.61	HCO_3_^−^	398.50
benzene	1.55	CO_3_^2−^	0
methylbenzene	1.63	Cl^−^	86.15
ethylbenzene	0.21	SO_4_^2−^	125.60
styrene	0.03	NO_3_^−^	13.78
m-xylene/p-xylene	0.26	Fe	6.51
o-xylene	0.13	Mn	3.48

## Supplementary Materials

The following are available online at https://www.mdpi.com/1660-4601/16/15/2752/s1, Figure S1. Change of geochemical parameters at monitoring wells 1 m downstream from injection wells. Ordinate: (a) pH; (b) oxidation-reduction potential (ORP); (c) electrical conductivity; (d) the concentration of SO42-(d); Figure S2. Concentration changes of pollutants at shallow monitoring well 1 m downstream from injection well. Ordinate: (a) the concentration of chlorinated hydrocarbons; (b) the concentration of BTEX. Inset: (a) amplified concentration change curves of 1,3-DCP, DCM and TCM; (b) amplified concentration change curves of EBZ, M/PX and OX. (d); Figure S3. Concentration changes of pollutants at deep monitoring well 1 m downstream from injection well. Ordinate: (a) the concentration of chlorinated hydrocarbons; (b) the concentration of BTEX. Inset: (a) amplified concentration change curves of 1,3-DCP, DCM and TCM; (b) amplified concentration change curves of EBZ, M/PX and OX. (d).

## Abbreviation

TCP	1,2,3-trichloropropane
ORP	oxidation−reduction potential
NZVIs	nanoscale zero-valent irons
ISCO	in situ chemical oxidation
PAHs	polycyclic aromatic hydrocarbons
VOCs	volatile organic compounds
FID	flame ionization detector
EC	electrical conductivity
TCE	trichloroethylene
1,2-DCP	1,2-dichloropropane
DCM	dichloromethane
BZ	benzene
EBZ	ethylbenzene
OX	o-xylene
BTEX	benzene, toluene, ethylbenzene, and xylene
(L)NAPL	(light) non-aqueous phase liquid
NZVZs	nanoscale zero-valent zincs
MTBE	methyl tert-butyl ether
PCBs	polychlorinated biphenyls
EDTA	ethylene diamine tetraacetic acid
GC	gas chromatograph
P&T/GC–MS	purge−trap/gas chromatograph mass
EDDS	ethylenediamine disuccinic acid
PCE	perchloroethylene
1,3-DCP	1,2-dichloropropane
TCM	trichloromethane
MBZ	methylbenzene
M/PX	m-xylene/p-xylene
