# Supplementary material for: In Situ Persulfate Oxidation of 1,2,3-Trichloropropane in Groundwater of North China Plain
Source: Int J Environ Res Public Health. 2019 Aug 1;16(15):2752. doi: 10.3390/ijerph16152752 (PMC6696075; doi:10.3390/ijerph16152752)
Supplement: Supplementary file 1 [file ijerph-16-02752-s001.pdf]

Supporting Information

In-Situ Persulfate Oxidation 1,2,3- Trichloropropane in Groundwater of North China Plain

Hui Li[1,2], Zhantao Han[1,2,*], Yong Qian[1,2], Xiangke Kong[1,2] and Ping Wang[1,2]

[1] Institute of Hydrogeology and Environmental Geology, Chinese Academy of Geological Sciences, Shijiazhuang 050061, China; lihui.107@163.com (H.L.); desertqy@163.com (Y.Q.); kongxiangke1987@163.com (X.K.); shuiwp@126.com (P.W.)

[2] Key Laboratory of Groundwater Remediation of Hebei Province and China Geological Survey, Shijiazhuang 050061, China

* Correspondence: hanzhantao1977@163.com; Tel.: +86-0311-67598806

Monitoring results from 1 m downstream wells

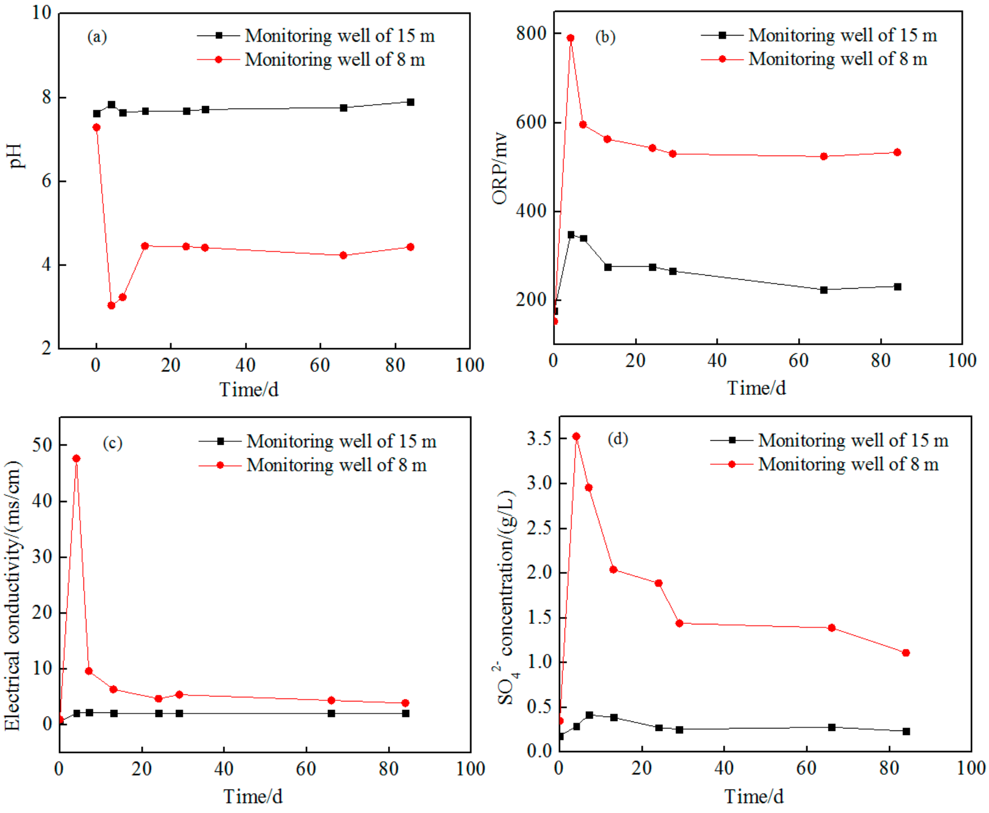

Figure S1. Change of geochemical parameters at monitoring wells 1 m downstream from injection wells. Ordinate: (a) pH; (b) oxidation-reduction potential (ORP); (c) electrical conductivity; (d) the concentration of SO_4^{2-}(d)

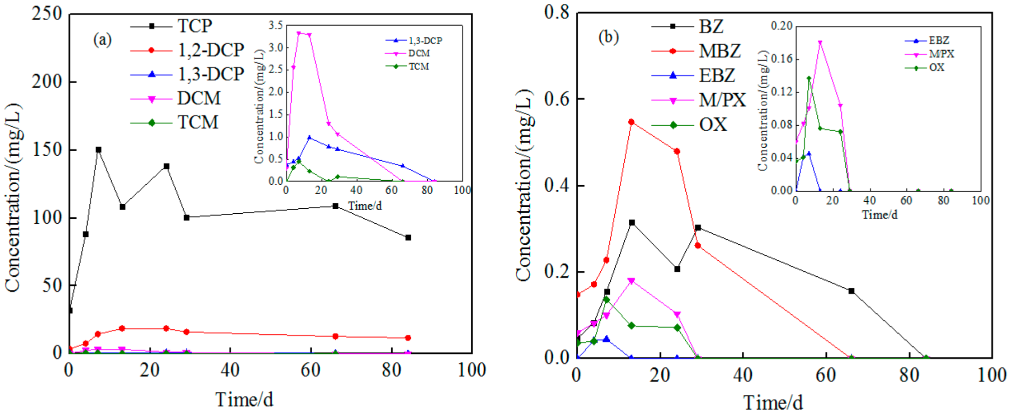

Figure S2. Concentration changes of pollutants at shallow monitoring well 1 m downstream from injection well. Ordinate: (a) the concentration of chlorinated hydrocarbons; (b) the concentration of BTEX. Inset: (a) amplified concentration change curves of 1,3-DCP, ~~DCP~~ DCM and TCM; (b) amplified concentration change curves of EBZ~~M~~, M/PX and OX. (d)

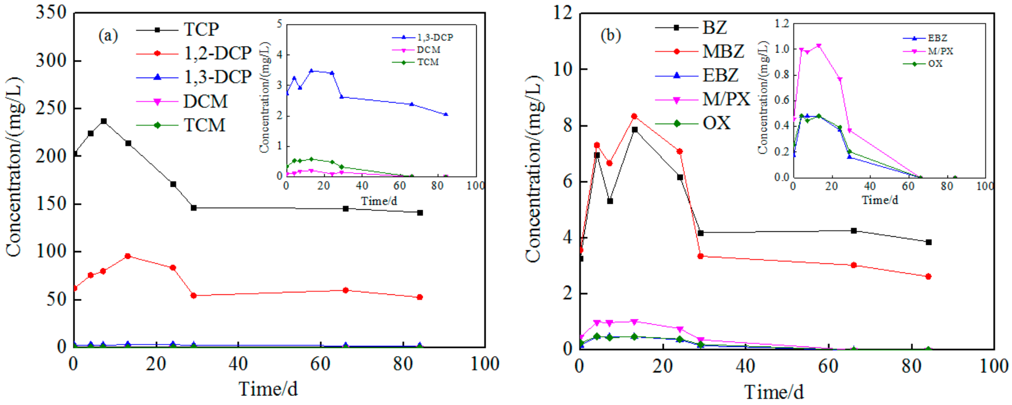

Figure S3. Concentration changes of pollutants at deep monitoring well 1 m downstream from injection well. Ordinate: (a) the concentration of chlorinated hydrocarbons; (b) the concentration of BTEX. Inset: (a) amplified concentration change curves of 1,3-DCP, ~~DCP~~ DCM and TCM; (b) amplified concentration change curves of ~~EBM~~EBZ, M/PX and OX. (d)
